# Least Squares Neural Network-Based Wireless E-Nose System Using an SnO_2_ Sensor Array

**DOI:** 10.3390/s18051446

**Published:** 2018-05-06

**Authors:** Areej Shahid, Jong-Hyeok Choi, Abu ul Hassan Sarwar Rana, Hyun-Seok Kim

**Affiliations:** Division of Electronics and Electrical Engineering, Dongguk University-Seoul, Seoul 04620, Korea; areejshahid.146@gmail.com (A.S.); yibee1226@naver.com (J.-H.C.); a.hassan.rana@gmail.com (A.u.H.S.R.)

**Keywords:** gas sensor array, pattern recognition, artificial neural network, least squares, concentration estimation

## Abstract

Over the last few decades, the development of the electronic nose (E-nose) for detection and quantification of dangerous and odorless gases, such as methane (CH_4_) and carbon monoxide (CO), using an array of SnO_2_ gas sensors has attracted considerable attention. This paper addresses sensor cross sensitivity by developing a classifier and estimator using an artificial neural network (ANN) and least squares regression (LSR), respectively. Initially, the ANN was implemented using a feedforward pattern recognition algorithm to learn the collective behavior of an array as the signature of a particular gas. In the second phase, the classified gas was quantified by minimizing the mean square error using LSR. The combined approach produced 98.7% recognition probability, with 95.5 and 94.4% estimated gas concentration accuracies for CH_4_ and CO, respectively. The classifier and estimator parameters were deployed in a remote microcontroller for the actualization of a wireless E-nose system.

## 1. Introduction

The detection, recognition, and concentration estimation of toxic gases, such as methane (CH_4_) and carbon monoxide (CO), remain a significant problem for rapidly growing industrial states. The inflammability and carcinogenic nature of these odorless gases demands fast, efficient, and reliable safety measures because exposure beyond certain concentrations can cause diseases and even death. The danger levels of CH_4_ and CO, as per international standards, have been summarized in Tables S1 and S2, respectively. Therefore, the installment of wireless monitoring devices at vulnerable fields and places, such as in coal mines, food quality assessment, disease diagnosis, gas storage plants, and petroleum industries, has grown vital for environmental control and disaster prevention [1,2,3,4].

Metal-oxide semiconductor-based gas sensors have been developed over the last few decades due to their high sensitivity, low power consumption, low cost, small size, and stability. Additional advantages include temperature controllability, on-chip integration facility, and the large number of gases these can detect [5]. Various oxides, including ZnO, Fe_2_O_3_, SnO_2_, Ga_2_O_3_, WO_3_, and TiO_2_, have shown responsivity to a range of gases such as H_2_, O_2_, NO, NH_3_, H_2_S, CO, CH_4_, HCHO, C_2_H_5_OH, C_2_H_2_, CH_3_SH, (CH_3_)_3_N, and other volatile organic compounds [6,7,8,9]. Many companies such as City Technology, MicroChem, Nissha FIS, Inc., and SparkFun Electronics provide these sensors in various commercialized forms [10,11,12].

The current paper utilizes SnO_2_-based commercial gas sensors for volatile combustible hydrocarbons, which change their electrical characteristics based on variations in atmospheric composition [13]. The design, applications, and material science of SnO_2_ sensors have been thoroughly investigated by many research groups [14,15,16]. Wang et al. discussed sensing mechanisms and influencing factors [17]. Although the sensors have high detection power, cross sensitivity requires a robust trained system that can classify target gases in a mixture with minimum ambiguity [18,19,20,21]. The SnO_2_ film also ages with time, humidity and temperature variations, and overexposure to target gases [22,23,24]. Thus, a self-adjustable scaling and calibration tool is required with a high precision concentration estimator.

Many previous studies have already addressed the gas identification and concentration estimation problems, including implementation of fuzzy logic designs, multilayer perceptron, artificial neural networks (ANN), and principal component analysis [25,26,27,28,29,30,31]. Support vector machines (SVMs) have been recently employed for a least squares approach to estimate gas concentration [32,33,34,35]. Varun et al. presented a conjugate gradient neural network (CGNN) based manhole gas detection system [36]. Various pattern recognition tools have been used for classification of odorless gases [37]. Srivastava introduced a mean and variance-based method for data transformation [38]. To cater for the instability of gas sensors, noise measurements and optimization approaches have been investigated [39,40,41]. Linear least squares and nonlinear SVM classifiers have been previously compared using a single electrolytic gas sensor [42].

This study exploited sensor array characteristics and mean centering prior to the application of two principal techniques: ANN and least squares regression (LSR). After preprocessing the sensor signals, the ANN classifier was designed on MATLAB^®^, providing better identification performance than raw data acquired from the sensors. The quality assurance of ANN showed 98.7% classification accuracy, which is a hallmark with the usage of cheap, cross sensitive, and aging sensors. Subsequent LSR estimated gas concentrations had 95.5 and 94.4% minimum accuracies, which is superior to conventional linear, polynomial, or logarithmic regression.

This paper is organized as follows. Section 2 describes the proposed electronic nose (E-nose) and experimental details. We provide a detailed overview of the data centering approach prior to ANN implementation for gas type classification, followed by the subsequent LSR to estimate the concentration. Section 3 discusses outcomes from this proposed approach. Finally, Section 4 summarizes the research findings and conclusions.

## 2. Materials and Methods

### 2.1. Experimental Setup

#### 2.1.1. Gas Sensor Setup

We employed an array of SnO_2_ gas sensors specialized for CH_4_ and CO detection. Variations in the metal-oxide semiconductor electrical properties arise from adsorption of gas molecules. Initially, oxygen from the air gets adsorbed onto the SnO_2_ surface and a transfer of electrons from SnO_2_ to oxygen takes place. This depletes electrons in the SnO_2_ surface region, increasing its resistance. In the presence of CH_4_ or CO, oxygen ions chemically react with injected gas particles, releasing electrons that transfer back to the near SnO_2_ surface. This decreases the material resistance and provides the sensor response [6,13,16].

A single sensor is not highly selective in its response but detects a wide range of compounds. Therefore, an array of different sensors is commonly employed to detect and identify gases, generating recognizable patterns for different analytes [7]. In particular, we employed commercially available MQ-4 and MQ-7 sensors due to their relatively high sensitivity for volatile organic gases [18,19]. Since the gas sensor response is severely affected by environmental changes, an auxiliary DHT11 sensor was included to monitor temperature and humidity [43]. Table 1 shows the sensor specifications used in the proposed E-nose system.

The chemisorption ability of SnO_2_ in the presence of detectable gases varies with respect to the type of analyte. The datasheets of MQ-4 and MQ-7 demonstrate the sensor resistance plot against the concentration of various gases [18,19]. It is clearly manifested that, in the presence of CH_4_ and CO, MQ-4 has higher sensitivity for CH_4_ whereas MQ-7 is more responsive to CO.

#### 2.1.2. System Setup

**Figure 1:**
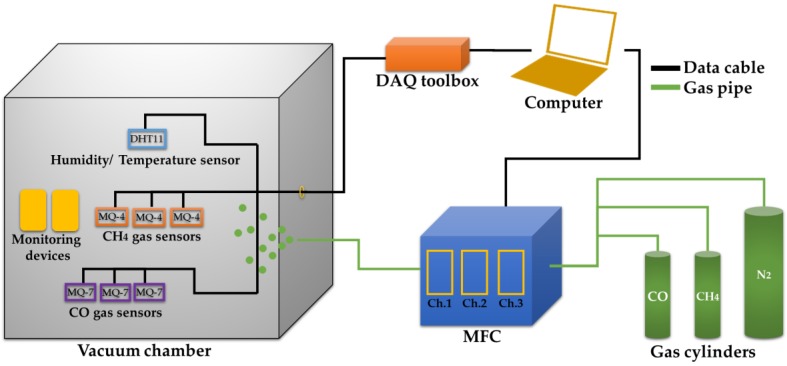
Experimental setup of an E-nose.

Figure 1 shows the hardware setup of the proposed E-nose system. After providing electrical connections, three each MQ-4 and MQ-7 sensors were placed in an experimental chamber with 100 L internal volume, which was a slave node. A vacuum pump removed the remaining gaseous contents in the chamber before and after each experiment. A mass flow controller (MFC) with a graphical user interface (GUI) was attached to CH_4_, CO and N_2_ gas cylinders. A data acquisition (DAQ) device with plug-and-play connectivity via RS232 protocol was used to acquire and log data, with inputs for gas, temperature, and humidity sensors. The MFC and DAQ were connected to the master node, which was a computer featured with prototyping, algorithm development, and code generation tools for fast and efficient real-time signal processing.

### 2.2. Sample Preparation

The MFC operated on standard cubic centimeter per minute (sccm) and standard liter per minute (slm) flow units, with selected flow rates 30 sccm, 6 sccm, and 10 slm for CH_4_, CO, and N_2_, respectively. Since the metal-oxide sensors require oxygen to function, ambient atmosphere was provided before injecting the target gases.

Owing to a plethora of health and environmental hazards associated with these gases, as described in Tables S1 and S2, the experimental range was set to 0–1200 ppm and 0–200 ppm for CH_4_ and CO, respectively. A total of 20 CH_4_ samples were tested for 24 concentrations ranging from 0 to 1200 ppm (50 ppm intervals), producing 480 data points; whereas 15 CO samples were tested for 20 concentrations ranging from 0 to 200 ppm (10 ppm intervals), producing 300 data points. Each data point constituted the analog outputs from all sensors in the array. An MFC GUI input control was used to automate the process. Both gases were injected in pure form, with equal time intervals and gas increments to analyze the sensor array collective response pattern. Temperature and humidity were maintained at 22 °C and 40% RH, respectively.

### 2.3. Data Acquisition and Preprocessing

A DAQ toolbox with a supporting GUI was employed to read, display, and log analog voltages from the six gas sensors and one auxiliary sensor. The sampling rate was set to one reading per second until the system was trained.

**Figure 2:**
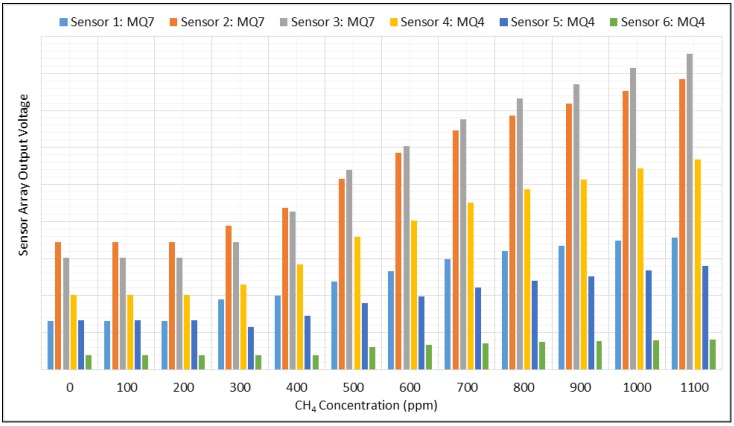
Variation of the output voltages of gas sensors in the presence of CH_4_.

The raw experimental data were first analyzed and inspected visually. Each sensor had initial air resistance, R_0_, that decreased to R_s_ in the presence of the detectable gas. R_0_ and R_s_ were different for each sensor, producing different output voltages from similar sensors, hence producing similar waveforms but different offsets and means, as shown in Figure 2.

Let X_i_ be the feature matrix of raw data acquired from the k = 1, 2, …, 6 sensors for i samples, where

(1) $$i=\{\begin{array}{c} 1,2,3,\ldots \ldots ,24\;\mathrm{for}\;{\mathrm{CH}}_{4}\;\mathrm{gas}\\ 1,2,3,\ldots \ldots ,20\;\mathrm{for}\;\mathrm{CO}\;\mathrm{gas} \end{array},$$

and

(2) $$X_{i}=\left[\begin{array}{cccccc} X_{11} & X_{12} & X_{13} & X_{14} & X_{15} & X_{16}\\ X_{21} & X_{22} & X_{23} & X_{24} & X_{25} & X_{26}\\ \ldots & \ldots & \ldots & \ldots & \ldots & \ldots \\ \ldots & \ldots & \ldots & \ldots & \ldots & \ldots \\ \ldots & \ldots & \ldots & \ldots & \ldots & \ldots \\ X_{ik} & X_{ik} & X_{ik} & X_{ik} & X_{ik} & X_{ik} \end{array}\right].$$

The sensors were scaled and centered around the same mean to increase E-nose accuracy. The standard deviation (S_d_) and mean (M) were calculated for six sensors. The raw data X_ik_ was normalized to get X_nk_ as per Equation (3):

X_nk_ = (X_ik_ − M)/S_d_, (3)

This results in the formation of normalized data matrix, X_n_, given by Equation (4):

(4) $$X_{n}=\left[\begin{array}{cccccc} X_{11} & X_{12} & X_{13} & X_{14} & X_{15} & X_{16}\\ X_{21} & X_{22} & X_{23} & X_{24} & X_{25} & X_{26}\\ \ldots & \ldots & \ldots & \ldots & \ldots & \ldots \\ \ldots & \ldots & \ldots & \ldots & \ldots & \ldots \\ \ldots & \ldots & \ldots & \ldots & \ldots & \ldots \\ X_{nk} & X_{nk} & X_{nk} & X_{nk} & X_{nk} & X_{nk} \end{array}\right].$$

This global method for sensor normalization sets the mean at the origin and variance within the data to 1. The data was further range scaled to set dynamic range to [0, 1]. The preprocessed sensor data correspond to i samples taken at different gas concentration levels. Thus, the target output vector T, encompassing the actual gas concentration values, is

(5) $$T=\left[\begin{array}{c} T_{1}\\ T_{2}\\ \ldots \\ \ldots \\ \ldots \\ T_{i} \end{array}\right].$$

### 2.4. Artificial Neural Network for Classification

Inspired by the biological nervous systems, ANNs were designed to process raw data or information using a vast network of neurons or nodes where all nodes are interconnected, and the signal path is called a synapse. Typically, these signals are real numbers and the nodes calculate outputs using linear or non–linear functions. The operations are performed on raw data in different layers, including an input layer, hidden layer, and output layer [44].

**Figure 3:**
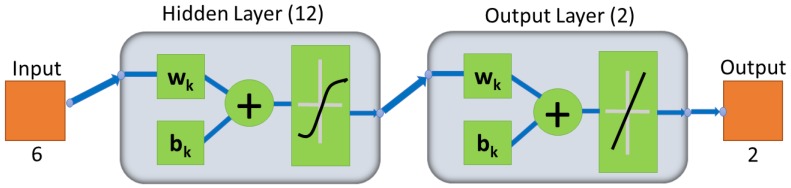
Three-layer ANN for gas classification.

Despite M and S_d_ variations, each sensor type has selective behavior, high responsivity, and similar waveforms to one particular gas, which were exploited in gas recognition neural networks [29,38,45]. The normalized signals from the gas sensor array were input to a feedforward ANN designed in MATLAB^®^ with six input neurons corresponding to the sensor arrays, twelve neurons in the hidden layers, and two output neurons corresponding to the identification result of both gases, as shown in Figure 3.

Considering the given data set as D = {(X_n_, T)}, where X_n_ is the normalized data for six sensors’ array and T is the target value, the input layer was fed with two data sets, one for each gas. A total of 500 data pairs from the experiments of each analyte were used. In these pairs, 300 belonged to CH_4_ experiments whereas 200 were from CO experiments. This data was divided randomly into training groups using 70% of the data, a validation group using 15% of the data, and a test group using 15% of the data. The six input neurons received X_n_ and transmitted it to hidden layer.

The hidden layer was composed of twelve neurons. Inside these neurons, every data pair was assigned weight (w_k_) and bias (b_k_) to input X_nk_ and then subjected to a function, g(X_n_), which was a non-linear hyperbolic tangent sigmoid function such that g(X_n_) approached T, as described in Equation (6):

(6) $$g\left(X_{n}\right)=\sum_{k=1}^{6}f\left[w_{k}\times \left(X_{\mathrm{nk}}\right)+b_{k}\right].$$

The network was trained iteratively to reassign w_k_ and b_k_ in each signal to produce the desired output value, which was the gas type. The network performance was assessed using a loss or cost factor (C) which was the mean square error (MSE). It is the average of squared errors between the target output (T) and the calculated network output (g(X_n_)) for N number of outputs. This factor was calculated using Equation (7):

(7) $$C=\mathrm{MSE}=\frac{1}{N}\sum_{1}^{N}{\left(g\left(X_{n}\right)-T\right)}^{2}.$$

The changes in updated values were dependent on the cost factor change with respect to previous w_k_ and b_k_ values. A decreasing error produced a smaller change from preceding values and vice versa. If the desired accuracy was not achieved, the algorithm updated w_k_ values in the next iteration as:

w_k+1_ = w_k_ + ἠ ∂C/∂w_k_, (8)

where ∂C/∂w_k_ is the gradient of error with respect to previous weights and ἠ is the learning rate that determines the amount of weight adjustment when the values are updated. A larger ἠ causes large changes in previous values and vice versa. The bias vector (b) followed the same upgradation criteria. The iteration count, in which all the vectors are used to update the w_k_ and b_k_ values, is called the epoch.

The momentum factor (Mu) determines the network training speed, and the optimal value can be found by trial and error. Table 2 shows minimum assigned performance factor and gradient, and maximum epochs, Mu, and validation checks to enhance network convergence and limit iterations. Training was terminated when any of the conditions were met.

The output layer was a linear transfer function to generate signals for the two output neurons. Each neuron detected the presence of one of the gases as a digital signal (0 or 1). The first neuron, for example, was reserved for CH_4_ detection and an output signal ~1 or ~0 meant the presence or absence of this gas, respectively. The final weights and biases for all neurons were stored in the network as w and b arrays, respectively, and exported to a microcontroller to provide a wireless and portable system.

### 2.5. Least Squares Regression to Estimate Concentrations

A concentration estimator was required after identifying the target gas for quantitative analysis of the analyte. However, the data was non-linear and, hence, unsuitable for simple mathematical models. Therefore, we used the Levenberg–Marquardt algorithm for the LSR of datum pairs [46,47,48]. An independent variable (X_n_), which was a preprocessed analog signal from sensors, and a dependent variable (T), which was a corresponding target gas concentration, constituted the LSR inputs. We devised an iterative procedure to produce a mathematical model using MATLAB^®^ fitting tools.

The goal was to derive coefficients (p) of a non-linear function (f) for each sensor that minimized the sum of squares of deviations from the target value. The mathematical representation of the problem statement is expressed as:

argmin (∑ [T − f(X_n_,p)]^2^). (9)

Since no single function is applicable to the whole sensor array, separate parameter vectors were calculated for each sensor, producing a set of six functions with their respective parameters,

(10) $$f(X_{n},p)=\{\begin{array}{c} f_{1}(X_{n},p_{1}),\;\mathrm{Sensor}\;1\\ f_{2}(X_{n},p_{2}),\;\mathrm{Sensor}\;2\\ f_{3}(X_{n},p_{3}),\;\mathrm{Sensor}\;3\\ f_{4}(X_{n},p_{4}),\;\mathrm{Sensor}\;4\\ f_{5}(X_{n},p_{5}),\;\mathrm{Sensor}\;5\\ f_{6}(X_{n},p_{6}),\;\mathrm{Sensor}\;6 \end{array},$$

where f_k_ was predefined, and the algorithm only calculated the parameters. As the sensor response pattern is unique for both gases therefore, two separate sets of functions and their parameters were devised. The type of model selected for LSR depended on the gas identified through ANN. The parameters were stored and used in the proposed wireless E-nose to estimate concentrations.

### 2.6. Emergency Alarm System

**Figure 4:**
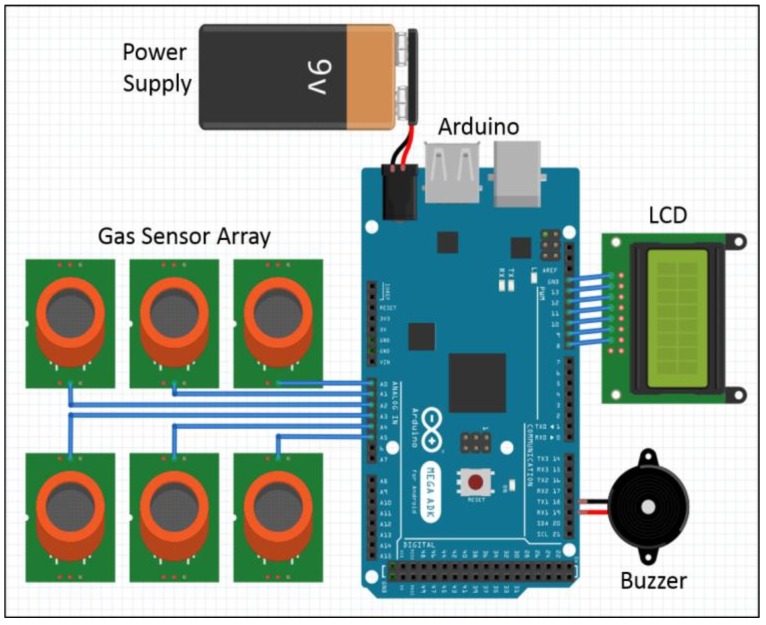
Proposed wireless E-nose system schematic.

Arduino Mega, an ATmega2560-based microcontroller, was connected to the gas sensor array with common power and ground connections [49]. The microcontroller connected to the computer via a USB and was programmed using C for gas classification and concentration estimation. The vectors M, S_d_, w, b, and p were stored in the microcontroller to implement the wireless E-nose and emergency alarm system. An LCD and buzzer were attached to monitor system output. Figure 4 shows the proposed E-nose system schematic.

**Figure 5:**
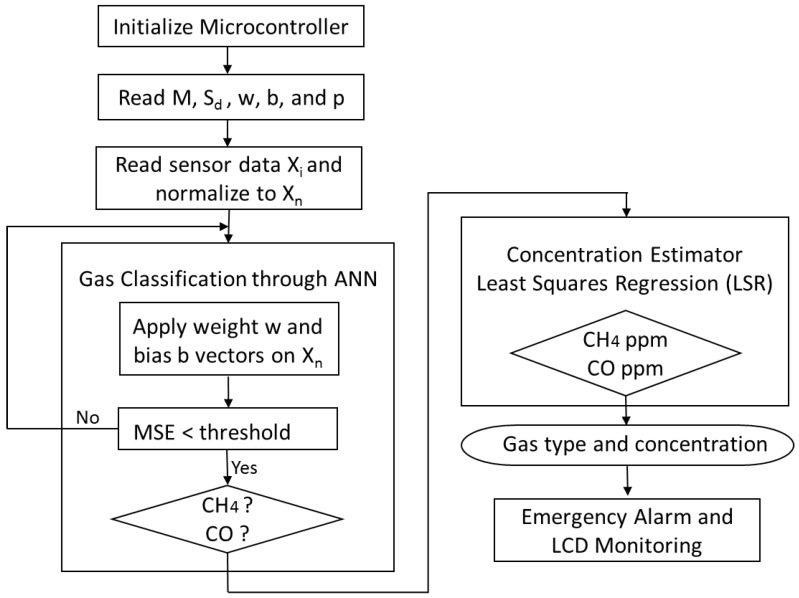
Proposed wireless E-nose flowchart.

The microcontroller reads all the stored vectors when initialized. The raw sensor data X_i_ is acquired in real-time and normalized. Gas presence was identified using ANN gas classification and concentration was then estimated using the LSR estimator. The results were displayed on the LCD and could be used as alarm inputs if the concentration exceeded dangerous levels. Figure 5 shows the proposed wireless E-nose flowchart.

## 3. Results and Discussion

### 3.1. Normalizing and Mean Centering

The MQ-4 detection range starts from 300 ppm of CH_4_, whereas that of MQ-7 starts from 20 ppm of CO. This is the minimum detection limit of the proposed E-nose system. Although there is a wide range of detectable gases for both sensors, this study mainly focused on higher selectivity for CH_4_ (MQ-4) and CO (MQ-7), which can be made more noticeable through normalization.

**Figure 6:**
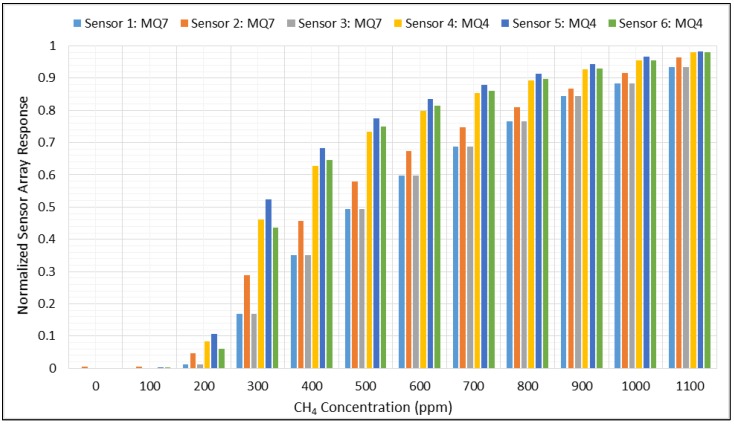
Normalized gas sensor array data in the presence of CH_4_.

Figure 6 summarizes the normalized data (from Equation (3)), highlighting the similar and overlapping patterns generated by the sensors under the same conditions.

### 3.2. Feature Extraction and Classification

The ANN consisted of an input layer that received normalized data from three MQ-4 and three MQ-7 sensors. The individual sensor patterns may not be selective, but the collective response of the whole array became predictable once the network was trained. The sensor array characteristic pattern in the presence of a particular gas is tantamount to a signature, which can be effectively learnt with sufficient training data.

**Figure 7:**
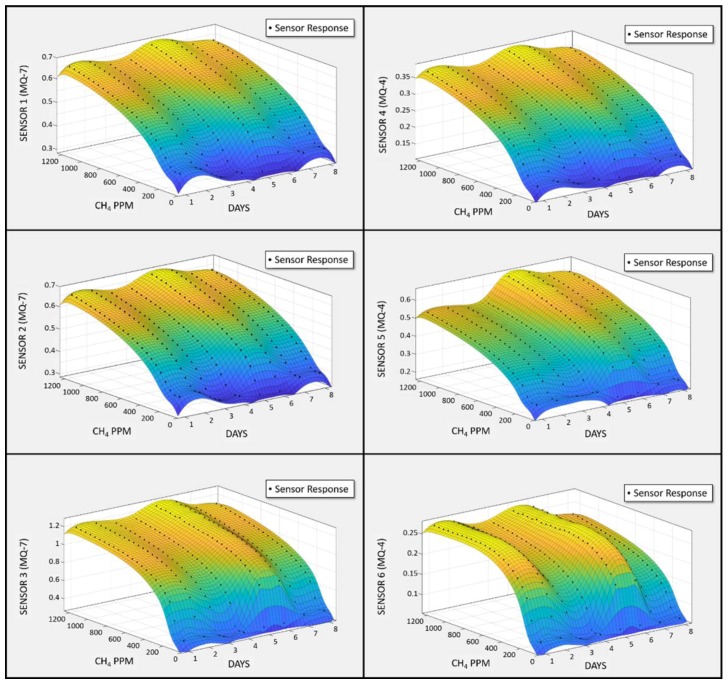
Output signal pattern of the gas sensor array in the presence of CH_4_.

**Figure 8:**
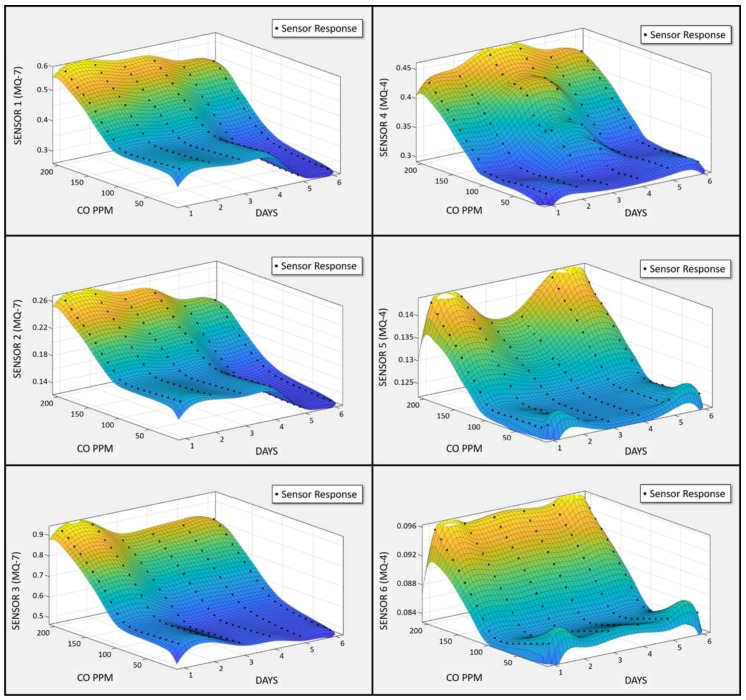
Output signal pattern of the gas sensor array in the presence of CO.

To visually analyze the pattern, 0–1200 ppm CH_4_ was injected in 50 ppm increments for each experiment. The recurrence of the same cycle each day for eight consecutive days generated a three-dimensional plot of sensor array behavior. Figure 7 shows the sensor array response plotted against gas concentration and the number of days when subjected to repetitive cycles of CH_4_. Similarly, there is a unique collective pattern for CO identification. The sensor array was subjected to an iterative procedure of CO gas injections ranging from 0 to 200 ppm for six consecutive days. The three-dimensional plot of the sensor array’s response against the number of days and CO concentration values is shown in Figure 8. MQ-4 and MQ-7 sensors are sensitive to both gases, but the higher selectivity of MQ-4 for CH_4_ and MQ-7 for CO is evident through steepness of the plots in Figure 7 and Figure 8, respectively. It should be noted that these plots are raw sensor data before normalization and, therefore, the dynamic range of all sensors has prominent diversity. Although the waveforms cannot be defined by linear, polynomial, or exponential expressions, the group responses successfully trained the ANN to recognize gas types.

### 3.3. Quality Assurance/Quality Control

The testing and validation results showed that the ANN distinguished gas signatures with improved performance after each iteration. This is because the MSE for the validation subset decreased from 1.4477 to 0.18 and, finally, to 0.09 in three consecutive training procedures. The final MSE value, which is a promising factor in assuring the quality of the classifier, was attained at epoch 3, thereby successfully identifying the gas type. To further inspect the validity for quality control, a confusion matrix for the classifier was obtained using 43 CH_4_ cases and 32 CO cases, as shown in Figure S1.

The matrix rows correspond to the network output or predicted gas class 1 and 2 for CH_4_ and CO, respectively, and the columns represent the target class or actual gas type. The first two diagonal cells show the number and percentage of correct classifications by the ANN, and the off-diagonal cells correspond to incorrectly classified gas types. The column on the far right shows the percentages of the classes which are classified correctly and incorrectly. Out of 43 CH_4_ cases, 42 (97.7%) were correctly predicted and only 1 (2.3%) was predicted as CO gas. Similarly, all 32 (100%) CO cases were correctly classified. The cell at the bottom right of the plot shows the overall accuracy of the classification as 98.7% with an error rate of 1.3%.

### 3.4. Estimated Gas Concentrations Using Least Squares Regression

The concentration was estimated after gas type classification using LSR. The dataset was split into training, validation, and test subsets using 70, 15 and 15% data respectively. A regression plot was created in MATLAB^®^ which showed the relationship between outputs of LSR and the desired target value. Here, the calculated gas concentration was denoted by Y and the target gas concentration by T.

**Figure 9:**
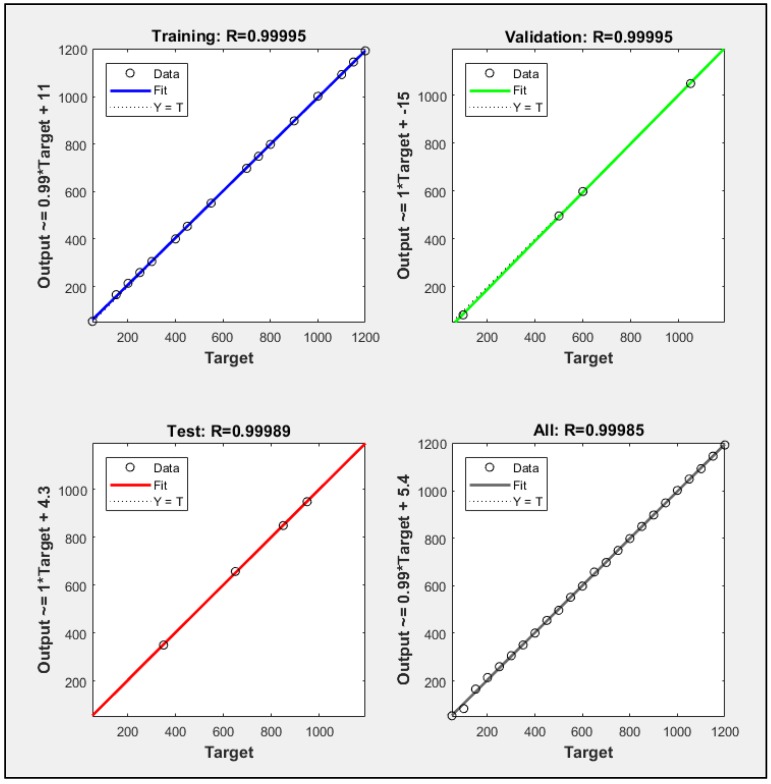
Training, validation, and test subset regressions for CH_4_.

**Figure 10:**
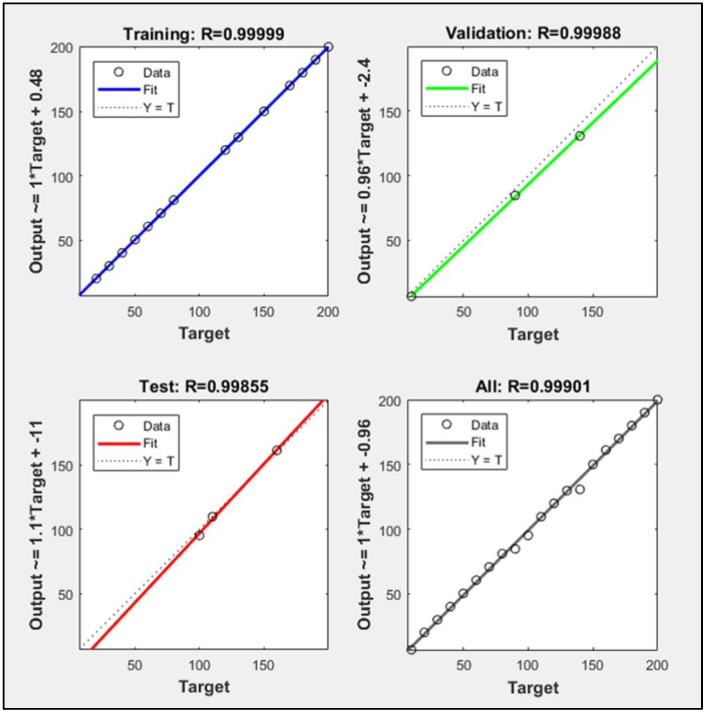
Training, validation, and test subset regression for CO.

Figure 9 and Figure 10 are regression plots which show CH_4_ and CO concentration estimate accuracy, respectively, where the horizontal axis is the target concentration and the vertical axis is the LSR-based estimated concentration. Ideally, LSR estimated values would be identical to the target values (i.e., Y ≡ T), which would generate a unitary correlation coefficient (R = 1), as shown by the dotted line in the regression plots. However, the original data (circles) does not fit perfectly, with several significant deviations from the dotted line. Such outliers are defined by the output-target relationship mentioned along the vertical axis. For example, a relatively larger magnitude of error in the validation subset of CO gas in Figure 10 is described by the expression (Output~ = 0.96*Target + −2.4) with a scale factor of 0.96 and an offset of −2.4.

Figure 9 shows that the CH_4_ concentration estimate achieved R = 99.995%, 99.995%, and 99.989% fit for the training, validation, and test groups, respectively. Therefore, an average of 99.985% match in the target and network output was achieved. Figure 10 shows that the CO concentration estimate achieved R = 99.999%, 99.988%, and 99.855% fit for the training, validation, and test groups, respectively. Hence, an average of 99.901% match in the target and network outputs was attained.

Table 3 shows the LSR-based concentration estimates for both gases. The minimum accuracy was 95.5 and 94.4% for CH_4_ and CO, respectively. The statistical significance of these experimental results is also evident in Figures S2 and S3, with sum of squared errors (SSE), root mean square error (RMSE), and error bars representing the standard deviation and standard error. However, the accuracy was attained for constant ambient conditions, the same sensor set, and a very short time span. In practice, sensor aging will become significant after prolonged exposure to high gas concentrations, inevitably impacting long-term accuracy. Also, any new set of sensors has to be calibrated and normalized before use. In these cases, retraining the algorithm would be a promising factor in ensuring E-nose efficiency.

The proposed E-nose system combines pattern recognition and LSR, delivering a high accuracy real-time monitoring system. Regression was greatly simplified by implementing the prior recognition procedure. The stored vectors as a result of classifier and estimator training were imported to the microcontroller and subjected to the same data sets. The real-time implementation means the proposed E-nose would be suitable for remote monitoring.

## 4. Conclusions

This study developed a wireless E-nose using an array of commercially available SnO_2_ gas sensors. An ANN was designed and employed using pattern recognition techniques to independently classify the presence of two toxic analytes, CH_4_ and CO, with 98.7% accuracy. A least squares regressor, trained as a gas concentration estimator, was implemented using the Levenberg–Marquardt algorithm. The trained network a had high recognition probability for both gases, and the subsequent LSR estimator achieved 95.5% and 94.4% minimum accuracies for CH_4_ and CO, respectively. The achieved model has a potential to detect various gas combinations by inserting a third output node in ANN to categorize “mixtures”, which will be addressed in our future study. Also, the same sensors and model can be retrained for other detectable gases such as LPG, H_2_, alcohol, and smoke. The derived ANN and LSR parameters were exported to a wireless microcontroller board. Hence, the proposition of two highly effective strategies to exploit the group behavior of the sensor array made it possible to actualize a real-time and precise wireless E-nose system.

## Figures and Tables

**Table 1:** Sensor specifications.

Sensor	Sensor Model	Detecting Materials ^1^	Range
CH_4_	MQ-4	CH_4_, LPG, H_2_, CO, Alcohol, smoke	300–10,000 ppm CH_4_
CO	MQ-7	CO, H_2_, LPG, CH_4_, Alcohol	10–500 ppm CO
Temperature and Humidity	DHT11	Temperature, Humidity	20–90% RH 0–50 °C

^1^ In order of decreasing sensitivity.

**Table 2:** ANN parameters for training.

Training Parameter	Assigned Value
Epochs	1000
Performance (MSE)	0.00
Gradient	1.00 × 10^−7^
Mu	1.00 × 10^10^
Validation Checks	20

**Table 3:** LSR-based gas concentration estimates.

CH_4_			CO		
Injected (ppm)	LSR Estimate (ppm)	Accuracy (%)	Injected (ppm)	LSR Estimate (ppm)	Accuracy (%)
50	51.3	97.3	10	10.4	95.8
100	102.0	97.9	20	21.1	94.7
150	156.0	95.9	30	28.9	96.6
200	193.0	96.5	40	37.7	94.4
250	241.9	96.7	50	47.9	95.9
300	313.3	95.5	60	61.9	96.6
350	358.1	97.7	70	68.0	97.1
400	409.5	97.6	80	81.3	98.3
450	455.9	98.7	90	91.9	97.7
500	490.9	98.2	100	102.0	98.0
550	555.1	99.1	110	108.3	98.4
600	611.0	98.1	120	118.9	99.1
650	650.0	100.0	130	131.9	98.4
700	699.7	99.9	140	137.9	98.5
750	750.1	99.9	150	151.7	98.8
800	795.9	99.4	160	162.8	98.1
850	850.1	99.9	170	167.8	98.7
900	899.9	99.9	180	185.5	96.9
950	946.9	99.6	190	193.8	97.9
1000	1002.9	99.7	200	197.8	98.9

## Supplementary Materials

The following are available online at http://www.mdpi.com/1424-8220/18/5/1446/s1. Table S1: CH_4_ concentration and safety level according to flammability risk, Table S2: CO concentration and associated health hazard according to international standards, Figure S1: Confusion matrix for ANN, Figure S2: Statistical evaluation of CH_4_ with SSE, RMSE, and error bars, Figure S3: Statistical evaluation of CO with SSE, RMSE, and error bars.
