# Supplementary material for: Least Squares Neural Network-Based Wireless E-Nose System Using an SnO2 Sensor Array
Source: Sensors (Basel). 2018 May 6;18(5):1446. doi: 10.3390/s18051446 (PMC5982671; doi:10.3390/s18051446)
Supplement: Supplementary file 1 [file sensors-18-01446-s001.pdf]

sensors

Supplementary Information

Least Squares Neural Network-based Wireless E-nose System using SnO_2 Sensor Array

Areej Shahid, Jong-Hyeok Choi, Abu ul Hassan Sarwar Rana and Hyun-Seok Kim*

Division of Electronics and Electrical Engineering, Dongguk University-Seoul, Seoul 04620, Korea
* Correspondence: hyunseokk@dongguk.edu, Tel: +82-2-2260-3996, Fax: +82-2-2277-8735

Table S1. CH_4 concentration and safety level according to flammability risk.

CH_4 Concentration (ppm)	Safety Level
0-199	Very good
200-399	Good
400-699	Normal
>700	Caution
>2000	Emergency Alarm

Table S2. CO concentration and associated health hazard according to international standards.

CO Concentration (ppm)	Health Effects
0	Fresh air
9	Maximum allowed indoor CO level (ASHRAE[1])
10-24	Carboxyhaemoglobin level in body can increase beyond 2.5% with long-term exposure
25	Max time weighted average exposure for 8 hours (ACGIH[2])
50	Maximum permissible exposure in workplace (OSHA[3])
100	Slight headache after 1-2 hours
200	Naseau, fagitue, and headache after 2-3 hours of exposure
400	Headache and nausea after 1-2 hours of exposure; life threatening after 3 hours
800	Naseau, headache, and dizziness after 45 minutes; collapse and unconsciousness after 1 hour of exposure; death within 2-3 hours
1000	Loss of consciousness after 1 hour of exposure
1600	Headache, nausea, and dizziness after 20 minutes of exposure; death within 1-2 hours
3200	Headache, nausea, and dizziness after 5-10 minutes; collapse and unconsciousness after 30 minutes of exposure; death within 1 hour
6400	Death within 30 minutes
12,800	Immediate physiological effects and loss of consciousness; death within 1-3 minutes of exposure

[1]American Society of Heating, Refrigerating, and Air Conditioning Engineers; [2]American Conference of Governmental Industrial Hygenists; [3]Occupational Safety and Health Administration

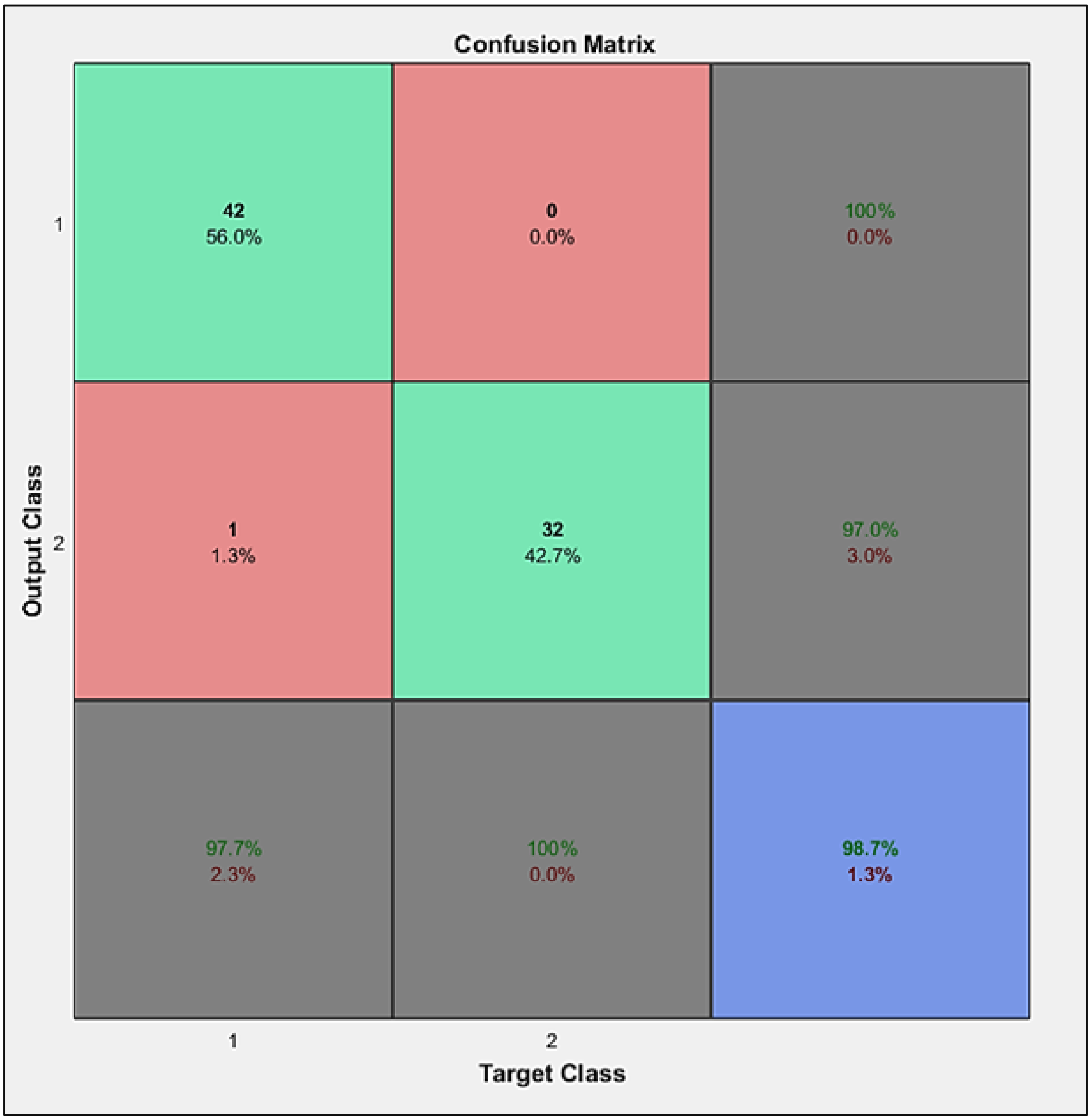

Figure S1. Confusion matrix for ANN.

Statistical evaluation of CH_4 and CO with SSE, RMSE, and error bars representing the value of uncertainty in terms of standard deviation and standard error have been shown in Figures S2 and S3, respectively. The error bars using standard deviation, which indicate the variability of values, are a range of values from mean minus standard deviation to the mean value of estimated data. Whereas, the error bars using standard error, which give an estimate of certainty of predicted values, are the 95% confidence interval (CI= point estimate ± 2 * standard error) of the estimated concentrations.

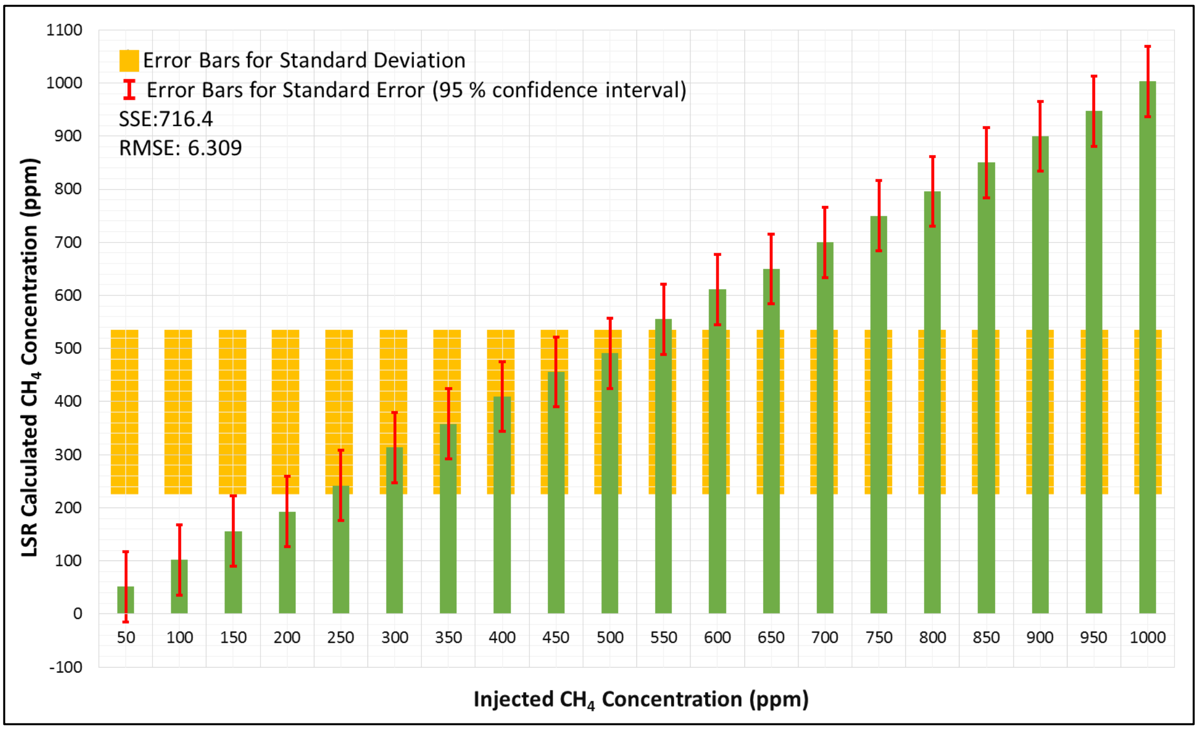

Figure S2. Statistical evaluation of CH_4 with SSE, RMSE, and error bars.

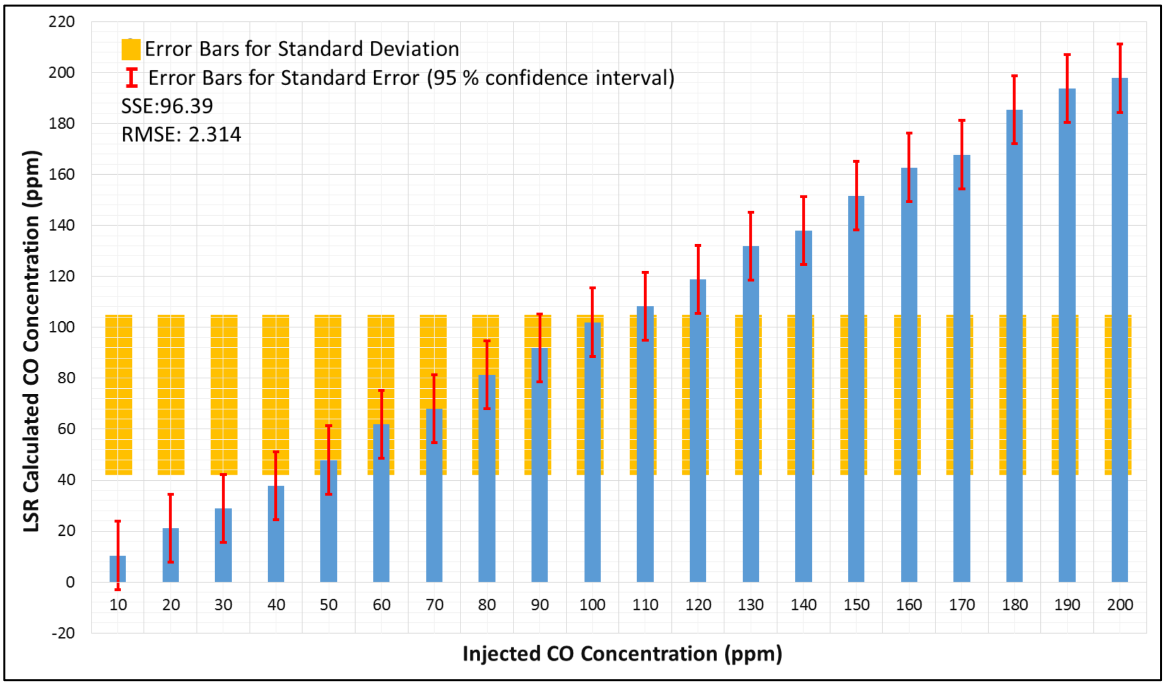

Figure S3. Statistical evaluation of CO with SSE, RMSE, and error bars.
